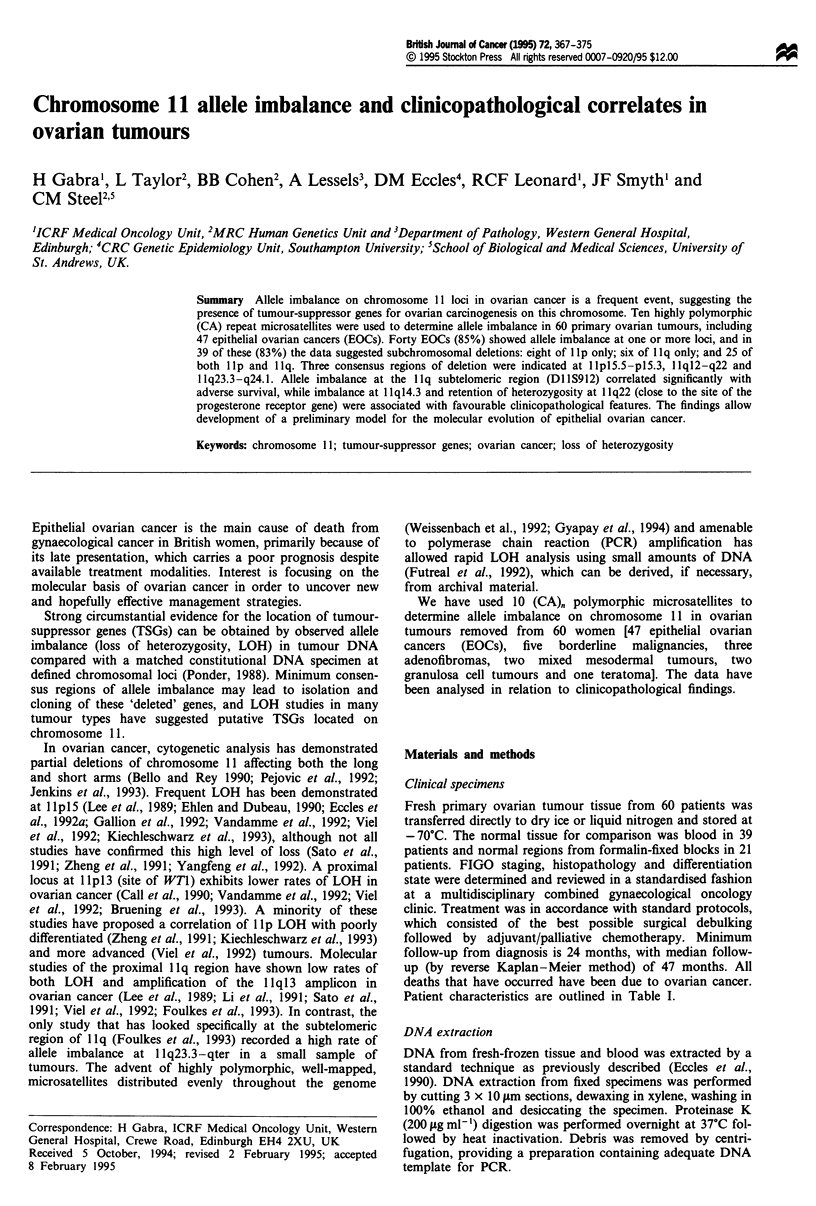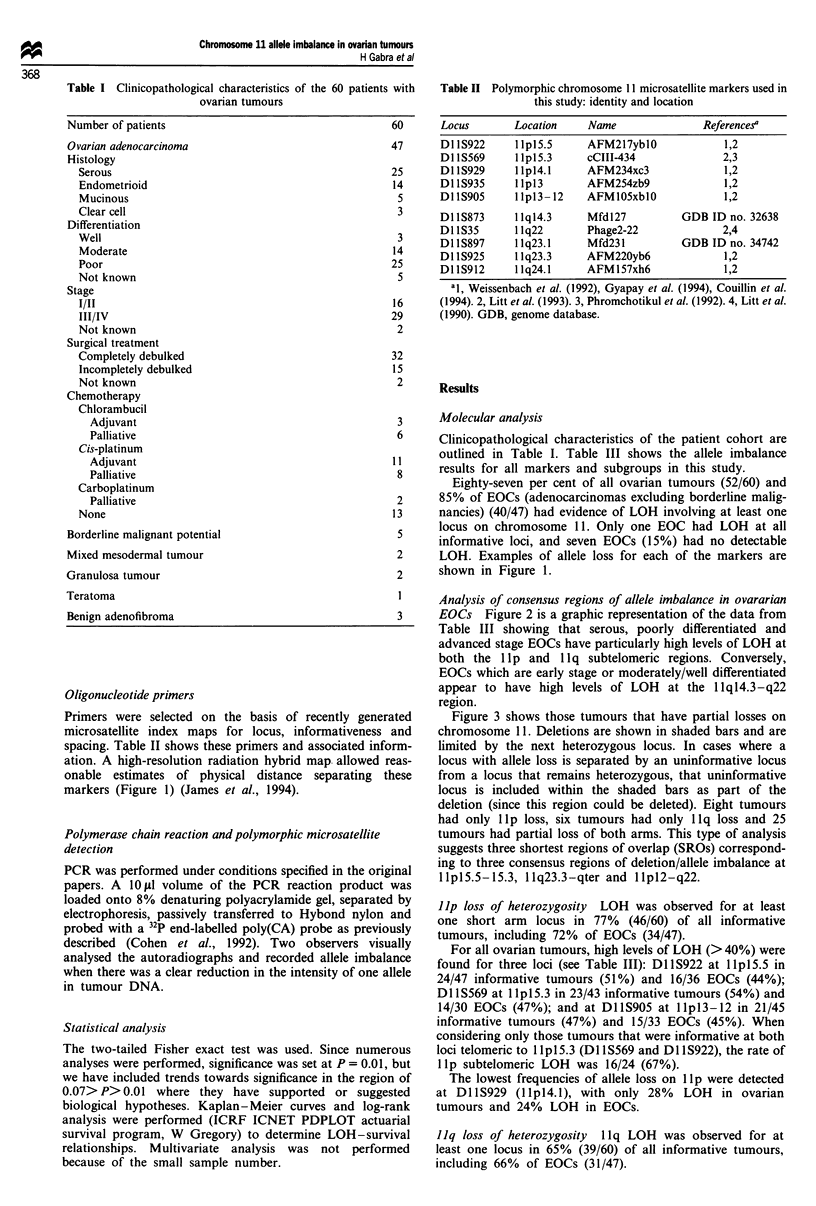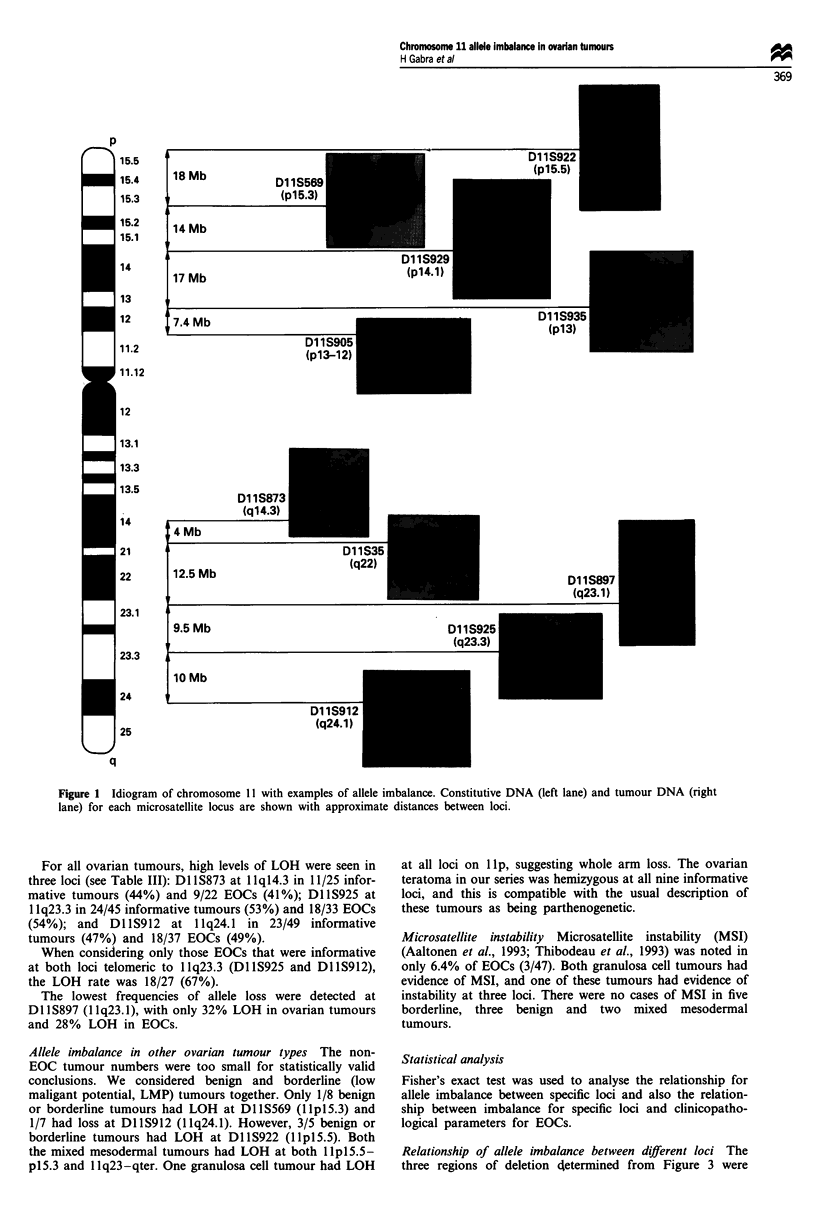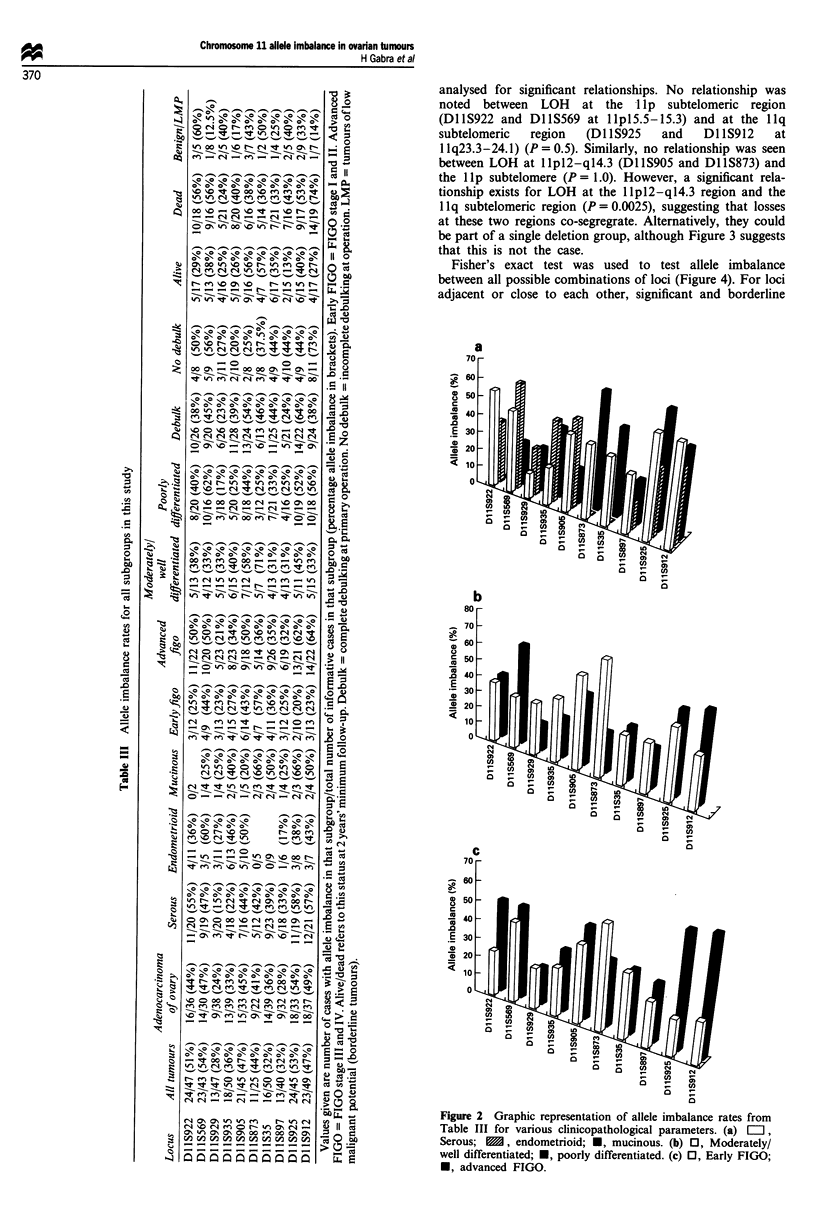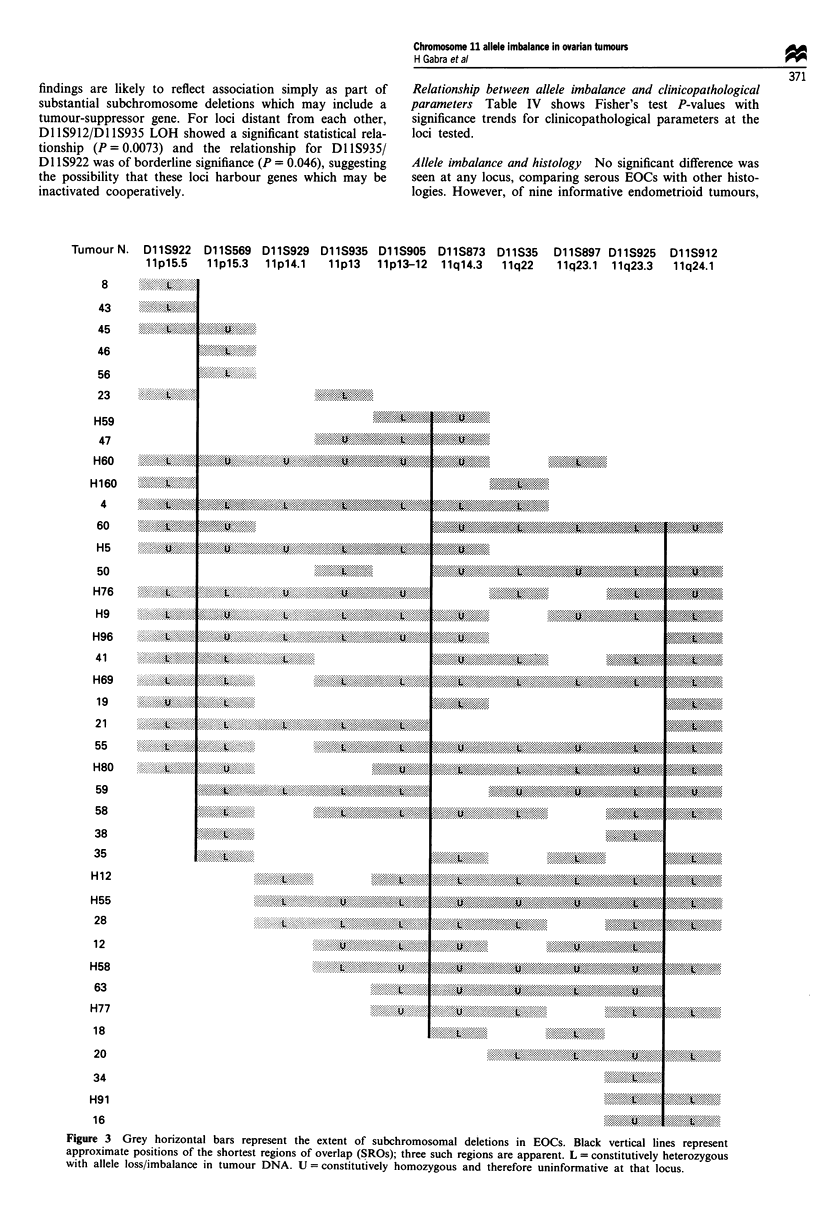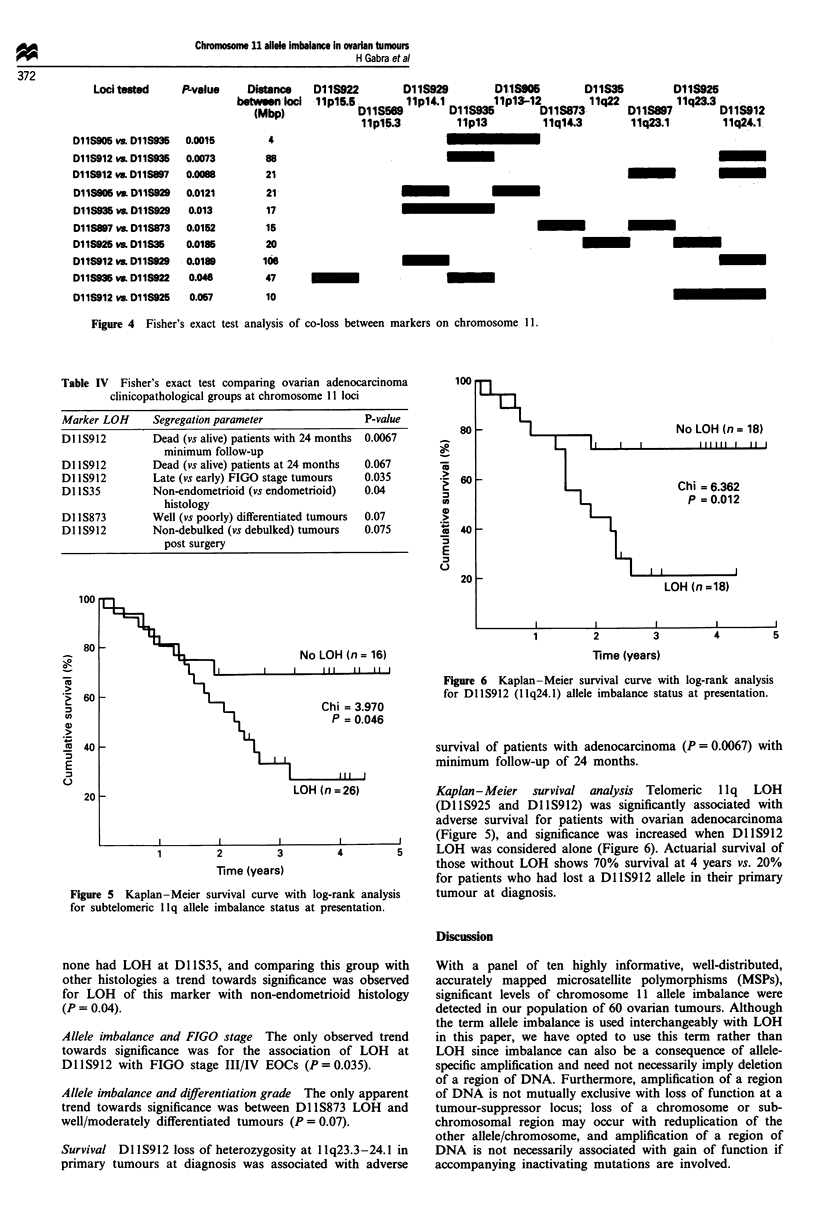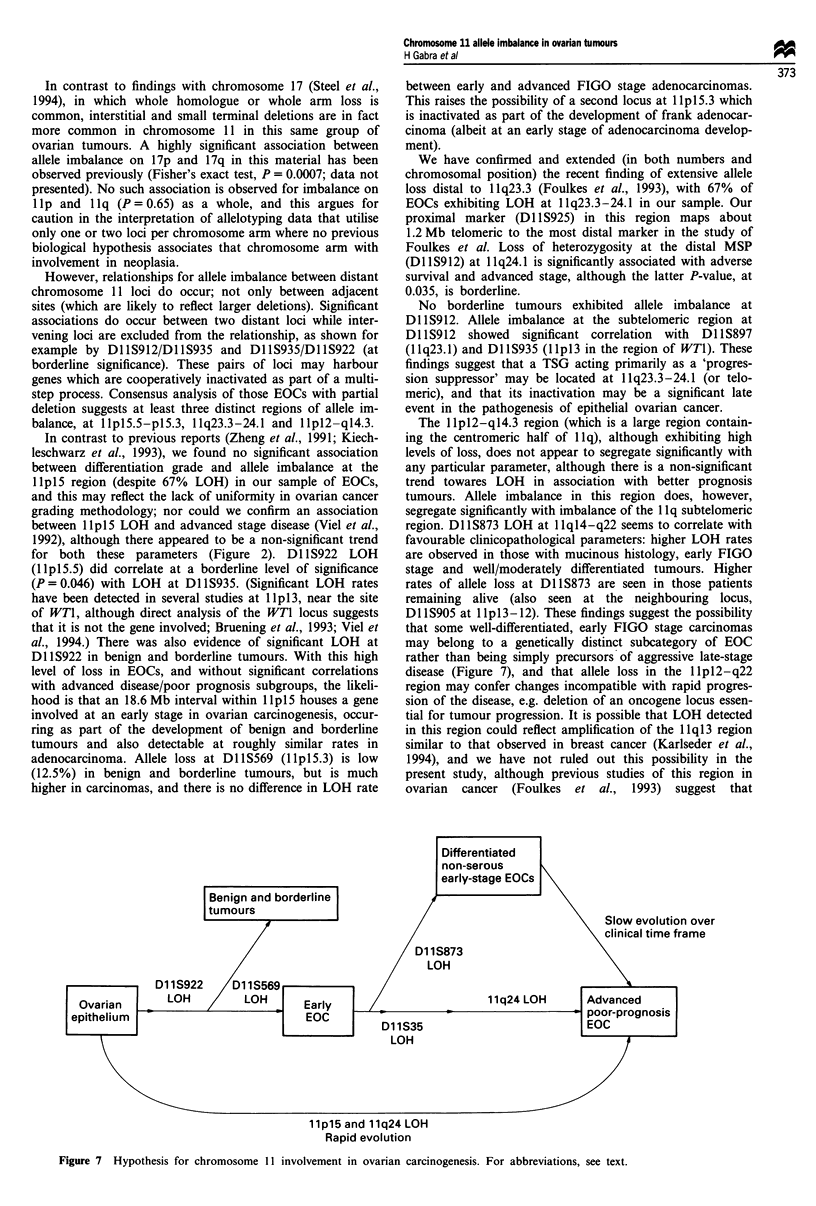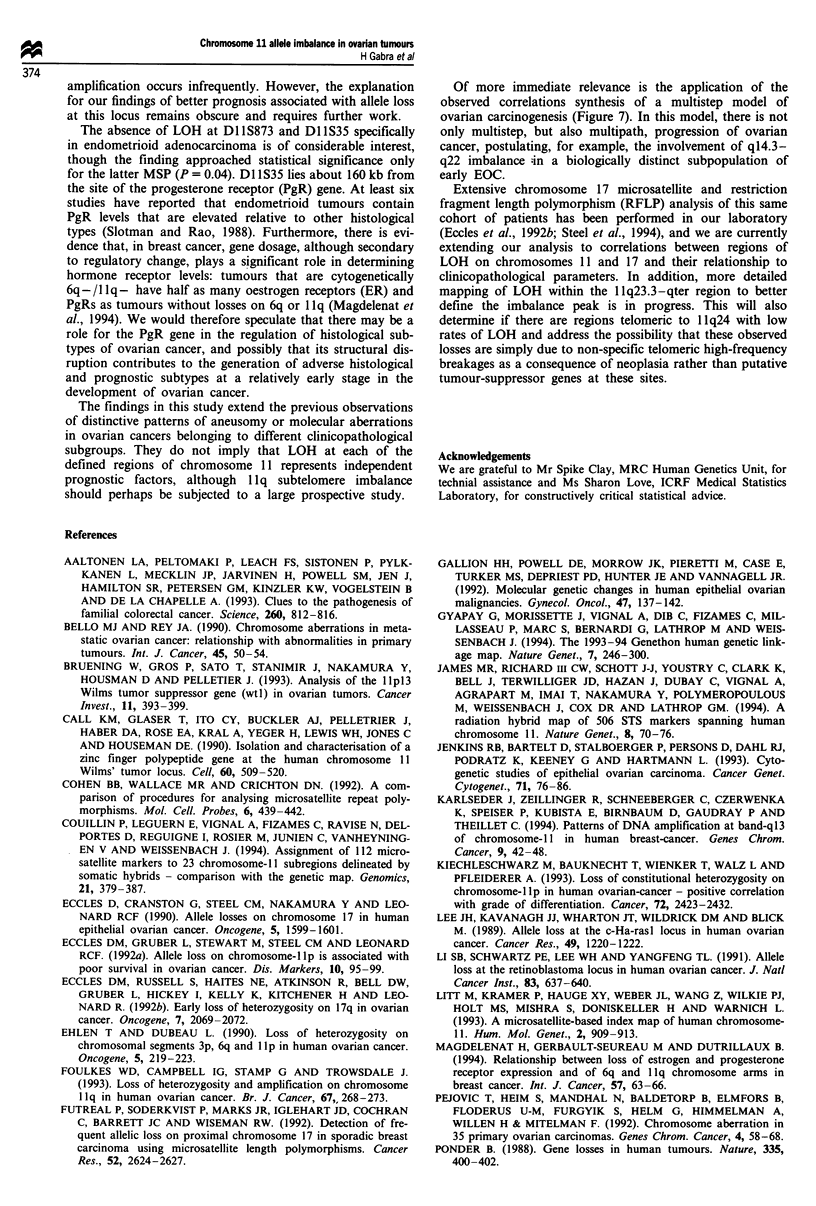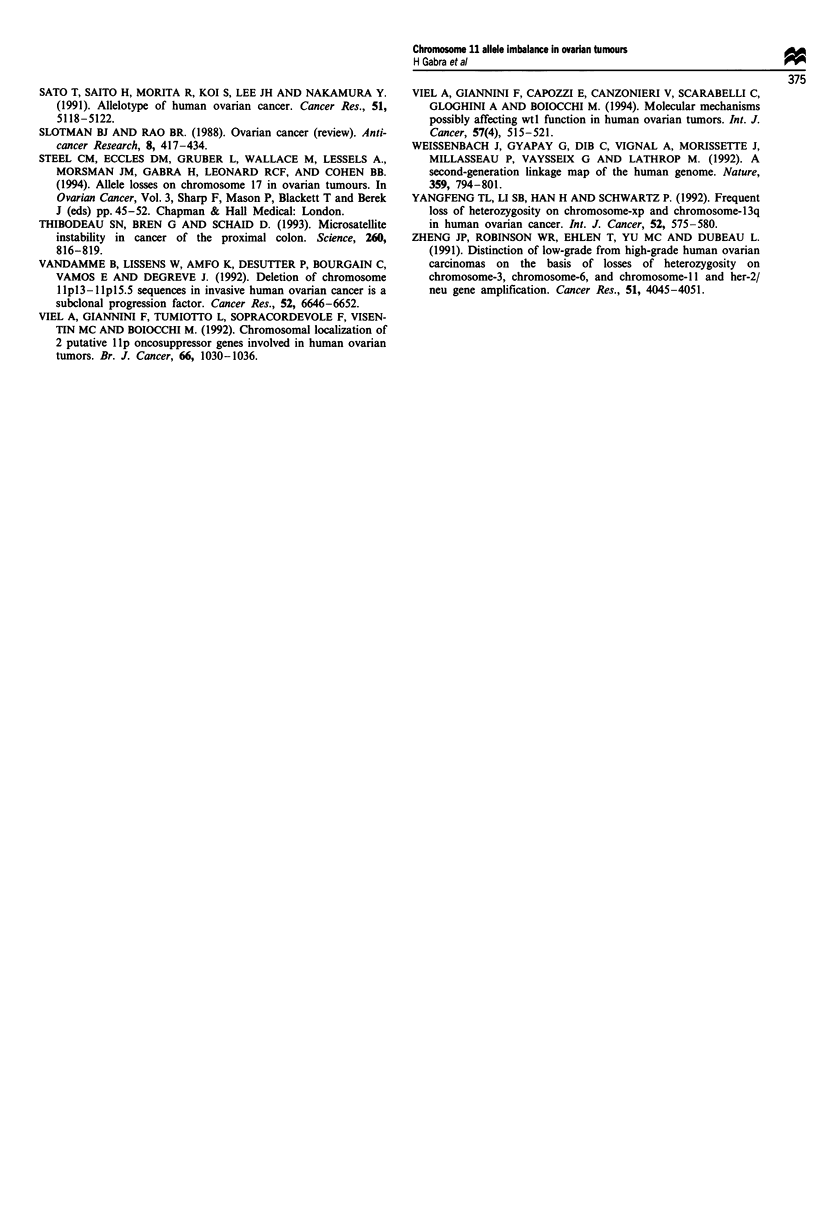# Chromosome 11 allele imbalance and clinicopathological correlates in ovarian tumours.

**DOI:** 10.1038/bjc.1995.340

**Published:** 1995-08

**Authors:** H. Gabra, L. Taylor, B. B. Cohen, A. Lessels, D. M. Eccles, R. C. Leonard, J. F. Smyth, C. M. Steel

**Affiliations:** ICRF Medical Oncology Unit, Western General Hospital, Edinburgh, UK.

## Abstract

**Images:**


British Journal of Cancer (1995) 72, 367-375
© 1995 Stockton Press All rights reserved 0007-0920/95 $12.00

### Chromosome 11 allele imbalance and clinicopathological correlates in ovarian tumours

H Gabra[1], L Taylor[2], BB Cohen[2], A Lessels[3], DM Eccles[4], RCF Leonard[1], JF Smyth[1] and CM Steel[2,5]

[1]*ICRF Medical Oncology Unit,* [2]*MRC Human Genetics Unit and* [3]*Department of Pathology, Western General Hospital, Edinburgh;* [4]*CRC Genetic Epidemiology Unit, Southampton University;* [5]*School of Biological and Medical Sciences, University of St. Andrews, UK.*

**Summary** Allele imbalance on chromosome 11 loci in ovarian cancer is a frequent event, suggesting the presence of tumour-suppressor genes for ovarian carcinogenesis on this chromosome. Ten highly polymorphic (CA) repeat microsatellites were used to determine allele imbalance in 60 primary ovarian tumours, including 47 epithelial ovarian cancers (EOCs). Forty EOCs (85%) showed allele imbalance at one or more loci, and in 39 of these (83%) the data suggested subchromosomal deletions: eight of 11p only; six of 11q only; and 25 of both 11p and 11q. Three consensus regions of deletion were indicated at 11p15.5-p15.3, 11q12-q22 and 11q23.3-q24.1. Allele imbalance at the 11q subtelomeric region (D11S912) correlated significantly with adverse survival, while imbalance at 11q14.3 and retention of heterozygosity at 11q22 (close to the site of the progesterone receptor gene) were associated with favourable clinicopathological features. The findings allow development of a preliminary model for the molecular evolution of epithelial ovarian cancer.

**Keywords:** chromosome 11; tumour-suppressor genes; ovarian cancer; loss of heterozygosity

Epithelial ovarian cancer is the main cause of death from gynaecological cancer in British women, primarily because of its late presentation, which carries a poor prognosis despite available treatment modalities. Interest is focusing on the molecular basis of ovarian cancer in order to uncover new and hopefully effective management strategies.

Strong circumstantial evidence for the location of tumour-suppressor genes (TSGs) can be obtained by observed allele imbalance (loss of heterozygosity, LOH) in tumour DNA compared with a matched constitutional DNA specimen at defined chromosomal loci (Ponder, 1988). Minimum consensus regions of allele imbalance may lead to isolation and cloning of these 'deleted' genes, and LOH studies in many tumour types have suggested putative TSGs located on chromosome 11.

In ovarian cancer, cytogenetic analysis has demonstrated partial deletions of chromosome 11 affecting both the long and short arms (Bello and Rey 1990; Pejovic *et al.*, 1992; Jenkins *et al.*, 1993). Frequent LOH has been demonstrated at 11p15 (Lee *et al.*, 1989; Ehlen and Dubeau, 1990; Eccles *et al.*, 1992*a*; Gallion *et al.*, 1992; Vandamme *et al.*, 1992; Viel *et al.*, 1992; Kiechleschwarz *et al.*, 1993), although not all studies have confirmed this high level of loss (Sato *et al.*, 1991; Zheng *et al.*, 1991; Yangfeng *et al.*, 1992). A proximal locus at 11p13 (site of *WT*1) exhibits lower rates of LOH in ovarian cancer (Call *et al.*, 1990; Vandamme *et al.*, 1992; Viel *et al.*, 1992; Bruening *et al.*, 1993). A minority of these studies have proposed a correlation of 11p LOH with poorly differentiated (Zheng *et al.*, 1991; Kiechleschwarz *et al.*, 1993) and more advanced (Viel *et al.*, 1992) tumours. Molecular studies of the proximal 11q region have shown low rates of both LOH and amplification of the 11q13 amplicon in ovarian cancer (Lee *et al.*, 1989; Li *et al.*, 1991; Sato *et al.*, 1991; Viel *et al.*, 1992; Foulkes *et al.*, 1993). In contrast, the only study that has looked specifically at the subtelomeric region of 11q (Foulkes *et al.*, 1993) recorded a high rate of allele imbalance at 11q23.3-qter in a small sample of tumours. The advent of highly polymorphic, well-mapped, microsatellites distributed evenly throughout the genome (Weissenbach et al., 1992; Gyapay *et al.*, 1994) and amenable to polymerase chain reaction (PCR) amplification has allowed rapid LOH analysis using small amounts of DNA (Futreal *et al.*, 1992), which can be derived, if necessary, from archival material.

We have used 10 $(CA)_n$ polymorphic microsatellites to determine allele imbalance on chromosome 11 in ovarian tumours removed from 60 women [47 epithelial ovarian cancers (EOCs), five borderline malignancies, three adenofibromas, two mixed mesodermal tumours, two granulosa cell tumours and one teratoma]. The data have been analysed in relation to clinicopathological findings.

### Materials and methods

### *Clinical specimens*

Fresh primary ovarian tumour tissue from 60 patients was transferred directly to dry ice or liquid nitrogen and stored at −70°C. The normal tissue for comparison was blood in 39 patients and normal regions from formalin-fixed blocks in 21 patients. FIGO staging, histopathology and differentiation state were determined and reviewed in a standardised fashion at a multidisciplinary combined gynaecological oncology clinic. Treatment was in accordance with standard protocols, which consisted of the best possible surgical debulking followed by adjuvant/palliative chemotherapy. Minimum follow-up from diagnosis is 24 months, with median follow-up (by reverse Kaplan-Meier method) of 47 months. All deaths that have occurred have been due to ovarian cancer. Patient characteristics are outlined in Table I.

### *DNA extraction*

DNA from fresh-frozen tissue and blood was extracted by a standard technique as previously described (Eccles *et al.*, 1990). DNA extraction from fixed specimens was performed by cutting 3 × 10 μm sections, dewaxing in xylene, washing in 100% ethanol and desiccating the specimen. Proteinase K (200 μg $ml^{-1}$) digestion was performed overnight at 37°C followed by heat inactivation. Debris was removed by centrifugation, providing a preparation containing adequate DNA template for PCR.

Correspondence: H Gabra, ICRF Medical Oncology Unit, Western General Hospital, Crewe Road, Edinburgh EH4 2XU, UK
Received 5 October, 1994; revised 2 February 1995; accepted 8 February 1995

**Table I** Clinicopathological characteristics of the 60 patients with ovarian tumours

| | |
|---|---|
| Number of patients | 60 |
| *Ovarian adenocarcinoma* | 47 |
| Histology | |
| Serous | 25 |
| Endometrioid | 14 |
| Mucinous | 5 |
| Clear cell | 3 |
| Differentiation | |
| Well | 3 |
| Moderate | 14 |
| Poor | 25 |
| Not known | 5 |
| Stage | |
| I/II | 16 |
| III/IV | 29 |
| Not known | 2 |
| Surgical treatment | |
| Completely debulked | 32 |
| Incompletely debulked | 15 |
| Not known | 2 |
| Chemotherapy | |
| Chlorambucil | |
| Adjuvant | 3 |
| Palliative | 6 |
| *Cis*-platinum | |
| Adjuvant | 11 |
| Palliative | 8 |
| Carboplatinum | |
| Palliative | 2 |
| None | 13 |
| Borderline malignant potential | 5 |
| Mixed mesodermal tumour | 2 |
| Granulosa tumour | 2 |
| Teratoma | 1 |
| Benign adenofibroma | 3 |

### Oligonucleotide primers

Primers were selected on the basis of recently generated microsatellite index maps for locus, informativeness and spacing. Table II shows these primers and associated information. A high-resolution radiation hybrid map allowed reasonable estimates of physical distance separating these markers (Figure 1) (James *et al.*, 1994).

### Polymerase chain reaction and polymorphic microsatellite detection

PCR was performed under conditions specified in the original papers. A 10 µl volume of the PCR reaction product was loaded onto 8% denaturing polyacrylamide gel, separated by electrophoresis, passively transferred to Hybond nylon and probed with a $^{32}P$ end-labelled poly(CA) probe as previously described (Cohen *et al.*, 1992). Two observers visually analysed the autoradiographs and recorded allele imbalance when there was a clear reduction in the intensity of one allele in tumour DNA.

### Statistical analysis

The two-tailed Fisher exact test was used. Since numerous analyses were performed, significance was set at $P = 0.01$, but we have included trends towards significance in the region of $0.07 > P > 0.01$ where they have supported or suggested biological hypotheses. Kaplan–Meier curves and log-rank analysis were performed (ICRF ICNET PDPLOT actuarial survival program, W Gregory) to determine LOH–survival relationships. Multivariate analysis was not performed because of the small sample number.

**Table II** Polymorphic chromosome 11 microsatellite markers used in this study: identity and location

| *Locus* | *Location* | *Name* | *References*[a] |
|---|---|---|---|
| D11S922 | 11p15.5 | AFM217yb10 | 1,2 |
| D11S569 | 11p15.3 | cCIII-434 | 2,3 |
| D11S929 | 11p14.1 | AFM234xc3 | 1,2 |
| D11S935 | 11p13 | AFM254zb9 | 1,2 |
| D11S905 | 11p13–12 | AFM105xb10 | 1,2 |
| D11S873 | 11q14.3 | Mfd127 | GDB ID no. 32638 |
| D11S35 | 11q22 | Phage2-22 | 2,4 |
| D11S897 | 11q23.1 | Mfd231 | GDB ID no. 34742 |
| D11S925 | 11q23.3 | AFM220yb6 | 1,2 |
| D11S912 | 11q24.1 | AFM157xh6 | 1,2 |

[a]1, Weissenbach *et al.* (1992), Gyapay *et al.* (1994), Couillin *et al.* (1994). 2, Litt *et al.* (1993). 3, Phromchotikul *et al.* (1992). 4, Litt *et al.* (1990). GDB, genome database.

### Results

### Molecular analysis

Clinicopathological characteristics of the patient cohort are outlined in Table I. Table III shows the allele imbalance results for all markers and subgroups in this study.

Eighty-seven per cent of all ovarian tumours (52/60) and 85% of EOCs (adenocarcinomas excluding borderline malignancies) (40/47) had evidence of LOH involving at least one locus on chromosome 11. Only one EOC had LOH at all informative loci, and seven EOCs (15%) had no detectable LOH. Examples of allele loss for each of the markers are shown in Figure 1.

*Analysis of consensus regions of allele imbalance in ovararian EOCs* Figure 2 is a graphic representation of the data from Table III showing that serous, poorly differentiated and advanced stage EOCs have particularly high levels of LOH at both the 11p and 11q subtelomeric regions. Conversely, EOCs which are early stage or moderately/well differentiated appear to have high levels of LOH at the 11q14.3–q22 region.

Figure 3 shows those tumours that have partial losses on chromosome 11. Deletions are shown in shaded bars and are limited by the next heterozygous locus. In cases where a locus with allele loss is separated by an uninformative locus from a locus that remains heterozygous, that uninformative locus is included within the shaded bars as part of the deletion (since this region could be deleted). Eight tumours had only 11p loss, six tumours had only 11q loss and 25 tumours had partial loss of both arms. This type of analysis suggests three shortest regions of overlap (SROs) corresponding to three consensus regions of deletion/allele imbalance at 11p15.5–15.3, 11q23.3–qter and 11p12–q22.

*11p loss of heterozygosity* LOH was observed for at least one short arm locus in 77% (46/60) of all informative tumours, including 72% of EOCs (34/47).

For all ovarian tumours, high levels of LOH (>40%) were found for three loci (see Table III): D11S922 at 11p15.5 in 24/47 informative tumours (51%) and 16/36 EOCs (44%); D11S569 at 11p15.3 in 23/43 informative tumours (54%) and 14/30 EOCs (47%); and at D11S905 at 11p13–12 in 21/45 informative tumours (47%) and 15/33 EOCs (45%). When considering only those tumours that were informative at both loci telomeric to 11p15.3 (D11S569 and D11S922), the rate of 11p subtelomeric LOH was 16/24 (67%).

The lowest frequencies of allele loss on 11p were detected at D11S929 (11p14.1), with only 28% LOH in ovarian tumours and 24% LOH in EOCs.

*11q loss of heterozygosity* 11q LOH was observed for at least one locus in 65% (39/60) of all informative tumours, including 66% of EOCs (31/47).

**Figure 1** Idiogram of chromosome 11 with examples of allele imbalance. Constitutive DNA (left lane) and tumour DNA (right lane) for each microsatellite locus are shown with approximate distances between loci.

For all ovarian tumours, high levels of LOH were seen in three loci (see Table III): D11S873 at 11q14.3 in 11/25 informative tumours (44%) and 9/22 EOCs (41%); D11S925 at 11q23.3 in 24/45 informative tumours (53%) and 18/33 EOCs (54%); and D11S912 at 11q24.1 in 23/49 informative tumours (47%) and 18/37 EOCs (49%).

When considering only those EOCs that were informative at both loci telomeric to 11q23.3 (D11S925 and D11S912), the LOH rate was 18/27 (67%).

The lowest frequencies of allele loss were detected at D11S897 (11q23.1), with only 32% LOH in ovarian tumours and 28% LOH in EOCs.

*Allele imbalance in other ovarian tumour types* The non-EOC tumour numbers were too small for statistically valid conclusions. We considered benign and borderline (low maligant potential, LMP) tumours together. Only 1/8 benign or borderline tumours had LOH at D11S569 (11p15.3) and 1/7 had loss at D11S912 (11q24.1). However, 3/5 benign or borderline tumours had LOH at D11S922 (11p15.5). Both the mixed mesodermal tumours had LOH at both 11p15.5–p15.3 and 11q23–qter. One granulosa cell tumour had LOH at all loci on 11p, suggesting whole arm loss. The ovarian teratoma in our series was hemizygous at all nine informative loci, and this is compatible with the usual description of these tumours as being parthenogenetic.

*Microsatellite instability* Microsatellite instability (MSI) (Aaltonen *et al.*, 1993; Thibodeau *et al.*, 1993) was noted in only 6.4% of EOCs (3/47). Both granulosa cell tumours had evidence of MSI, and one of these tumours had evidence of instability at three loci. There were no cases of MSI in five borderline, three benign and two mixed mesodermal tumours.

### *Statistical analysis*

Fisher's exact test was used to analyse the relationship for allele imbalance between specific loci and also the relationship between imbalance for specific loci and clinicopathological parameters for EOCs.

*Relationship of allele imbalance between different loci* The three regions of deletion determined from Figure 3 were

**Table III** Allele imbalance rates for all subgroups in this study

| Locus | All tumours | Adenocarcinoma of ovary | Serous | Endometrioid | Mucinous | Early figo | Advanced figo | Moderately/ well differentiated | Poorly differentiated | Debulk | No debulk | Alive | Dead | Benign/LMP |
|---|---|---|---|---|---|---|---|---|---|---|---|---|---|---|
| D11S922 | 24/47 (51%) | 16/36 (44%) | 11/20 (55%) | 4/11 (36%) | 0/2 | 3/12 (25%) | 11/22 (50%) | 5/13 (38%) | 8/20 (40%) | 10/26 (38%) | 4/8 (50%) | 5/17 (29%) | 10/18 (56%) | 3/5 (60%) |
| D11S569 | 23/43 (54%) | 14/30 (47%) | 9/19 (47%) | 3/5 (60%) | 1/4 (25%) | 4/9 (44%) | 10/20 (50%) | 4/12 (33%) | 10/16 (62%) | 9/20 (45%) | 5/9 (56%) | 5/13 (38%) | 9/16 (56%) | 1/8 (12.5%) |
| D11S929 | 13/47 (28%) | 9/38 (24%) | 3/20 (15%) | 3/11 (27%) | 1/4 (25%) | 3/13 (23%) | 5/23 (21%) | 5/15 (33%) | 3/18 (17%) | 6/26 (23%) | 3/11 (27%) | 4/16 (25%) | 5/21 (24%) | 2/5 (40%) |
| D11S935 | 18/50 (36%) | 13/39 (33%) | 4/18 (22%) | 6/13 (46%) | 2/5 (40%) | 4/15 (27%) | 8/23 (34%) | 6/15 (40%) | 5/20 (25%) | 11/28 (39%) | 2/10 (20%) | 5/19 (26%) | 8/20 (40%) | 1/6 (17%) |
| D11S905 | 21/45 (47%) | 15/33 (45%) | 7/16 (44%) | 5/10 (50%) | 1/5 (20%) | 6/14 (43%) | 9/18 (50%) | 7/12 (58%) | 8/18 (44%) | 13/24 (54%) | 2/8 (25%) | 9/16 (56%) | 6/16 (38%) | 3/7 (43%) |
| D11S873 | 11/25 (44%) | 9/22 (41%) | 5/12 (42%) | 0/5 | 2/3 (66%) | 4/7 (57%) | 5/14 (36%) | 5/7 (71%) | 3/12 (25%) | 6/13 (46%) | 3/8 (37.5%) | 4/7 (57%) | 5/14 (36%) | 1/2 (50%) |
| D11S35 | 16/50 (32%) | 14/39 (36%) | 9/23 (39%) | 0/9 | 2/4 (50%) | 4/11 (36%) | 9/26 (35%) | 4/13 (31%) | 7/21 (33%) | 11/25 (44%) | 4/9 (44%) | 6/17 (35%) | 7/21 (33%) | 1/4 (25%) |
| D11S897 | 13/40 (32%) | 9/32 (28%) | 6/18 (33%) | 1/6 (17%) | 1/4 (25%) | 3/12 (25%) | 6/19 (32%) | 4/13 (31%) | 4/16 (25%) | 5/21 (24%) | 4/10 (44%) | 2/15 (13%) | 7/16 (43%) | 2/5 (40%) |
| D11S925 | 24/45 (53%) | 18/33 (54%) | 11/19 (58%) | 3/8 (38%) | 2/3 (66%) | 2/10 (20%) | 13/21 (62%) | 5/11 (45%) | 10/19 (52%) | 14/22 (64%) | 4/9 (44%) | 6/15 (40%) | 9/17 (53%) | 2/9 (33%) |
| D11S912 | 23/49 (47%) | 18/37 (49%) | 12/21 (57%) | 3/7 (43%) | 2/4 (50%) | 3/13 (23%) | 14/22 (64%) | 5/15 (33%) | 10/18 (56%) | 9/24 (38%) | 8/11 (73%) | 4/17 (27%) | 14/19 (74%) | 1/7 (14%) |

Values given are number of cases with allele imbalance in that subgroup/total number of informative cases in that subgroup (percentage allele imbalance in brackets). Early FIGO = FIGO stage I and II. Advanced FIGO = FIGO stage III and IV. Alive/dead refers to this status at 2 years' minimum follow-up. Debulk = complete debulking at primary operation. No debulk = incomplete debulking at operation. LMP = tumours of low malignant potential (borderline tumours).

analysed for significant relationships. No relationship was noted between LOH at the 11p subtelomeric region (D11S922 and D11S569 at 11p15.5–15.3) and at the 11q subtelomeric region (D11S925 and D11S912 at 11q23.3–24.1) ($P = 0.5$). Similarly, no relationship was seen between LOH at 11p12–q14.3 (D11S905 and D11S873) and the 11p subtelomere ($P = 1.0$). However, a significant relationship exists for LOH at the 11p12–q14.3 region and the 11q subtelomeric region ($P = 0.0025$), suggesting that losses at these two regions co-segregrate. Alternatively, they could be part of a single deletion group, although Figure 3 suggests that this is not the case.

Fisher's exact test was used to test allele imbalance between all possible combinations of loci (Figure 4). For loci adjacent or close to each other, significant and borderline

**Figure 2** Graphic representation of allele imbalance rates from Table III for various clinicopathological parameters. (**a**) □, Serous; ▨, endometrioid; ■, mucinous. (**b**) □, Moderately/well differentiated; ■, poorly differentiated. (**c**) □, Early FIGO; ■, advanced FIGO.

findings are likely to reflect association simply as part of substantial subchromosome deletions which may include a tumour-suppressor gene. For loci distant from each other, D11S912/D11S935 LOH showed a significant statistical relationship ($P = 0.0073$) and the relationship for D11S935/D11S922 was of borderline signifiance ($P = 0.046$), suggesting the possibility that these loci harbour genes which may be inactivated cooperatively.

*Relationship between allele imbalance and clinicopathological parameters* Table IV shows Fisher's test $P$-values with significance trends for clinicopathological parameters at the loci tested.

*Allele imbalance and histology* No significant difference was seen at any locus, comparing serous EOCs with other histologies. However, of nine informative endometrioid tumours,

**Figure 3** Grey horizontal bars represent the extent of subchromosomal deletions in EOCs. Black vertical lines represent approximate positions of the shortest regions of overlap (SROs); three such regions are apparent. L = constitutively heterozygous with allele loss/imbalance in tumour DNA. U = constitutively homozygous and therefore uninformative at that locus.

**Figure 4** Fisher's exact test analysis of co-loss between markers on chromosome 11.

**Table IV** Fisher's exact test comparing ovarian adenocarcinoma clinicopathological groups at chromosome 11 loci

| *Marker LOH* | *Segregation parameter* | P-*value* |
|---|---|---|
| D11S912 | Dead (*vs* alive) patients with 24 months minimum follow-up | 0.0067 |
| D11S912 | Dead (*vs* alive) patients at 24 months | 0.067 |
| D11S912 | Late (*vs* early) FIGO stage tumours | 0.035 |
| D11S35 | Non-endometrioid (*vs* endometrioid) histology | 0.04 |
| D11S873 | Well (*vs* poorly) differentiated tumours | 0.07 |
| D11S912 | Non-debulked (*vs* debulked) tumours post surgery | 0.075 |

**Figure 5** Kaplan–Meier survival curve with log-rank analysis for subtelomeric 11q allele imbalance status at presentation.

none had LOH at D11S35, and comparing this group with other histologies a trend towards significance was observed for LOH of this marker with non-endometrioid histology ($P = 0.04$).

*Allele imbalance and FIGO stage* The only observed trend towards significance was for the association of LOH at D11S912 with FIGO stage III/IV EOCs ($P = 0.035$).

*Allele imbalance and differentiation grade* The only apparent trend towards significance was between D11S873 LOH and well/moderately differentiated tumours ($P = 0.07$).

*Survival* D11S912 loss of heterozygosity at 11q23.3–24.1 in primary tumours at diagnosis was associated with adverse

**Figure 6** Kaplan–Meier survival curve with log-rank analysis for D11S912 (11q24.1) allele imbalance status at presentation.

survival of patients with adenocarcinoma ($P = 0.0067$) with minimum follow-up of 24 months.

*Kaplan–Meier survival analysis* Telomeric 11q LOH (D11S925 and D11S912) was significantly associated with adverse survival for patients with ovarian adenocarcinoma (Figure 5), and significance was increased when D11S912 LOH was considered alone (Figure 6). Actuarial survival of those without LOH shows 70% survival at 4 years *vs*. 20% for patients who had lost a D11S912 allele in their primary tumour at diagnosis.

### Discussion

With a panel of ten highly informative, well-distributed, accurately mapped microsatellite polymorphisms (MSPs), significant levels of chromosome 11 allele imbalance were detected in our population of 60 ovarian tumours. Although the term allele imbalance is used interchangeably with LOH in this paper, we have opted to use this term rather than LOH since imbalance can also be a consequence of allele-specific amplification and need not necessarily imply deletion of a region of DNA. Furthermore, amplification of a region of DNA is not mutually exclusive with loss of function at a tumour-suppressor locus; loss of a chromosome or sub-chromosomal region may occur with reduplication of the other allele/chromosome, and amplification of a region of DNA is not necessarily associated with gain of function if accompanying inactivating mutations are involved.

In contrast to findings with chromosome 17 (Steel et al., 1994), in which whole homologue or whole arm loss is common, interstitial and small terminal deletions are in fact more common in chromosome 11 in this same group of ovarian tumours. A highly significant association between allele imbalance on 17p and 17q in this material has been observed previously (Fisher's exact test, $P = 0.0007$; data not presented). No such association is observed for imbalance on 11p and 11q ($P = 0.65$) as a whole, and this argues for caution in the interpretation of allelotyping data that utilise only one or two loci per chromosome arm where no previous biological hypothesis associates that chromosome arm with involvement in neoplasia.

However, relationships for allele imbalance between distant chromosome 11 loci do occur; not only between adjacent sites (which are likely to reflect larger deletions). Significant associations do occur between two distant loci while intervening loci are excluded from the relationship, as shown for example by D11S912/D11S935 and D11S935/D11S922 (at borderline significance). These pairs of loci may harbour genes which are cooperatively inactivated as part of a multistep process. Consensus analysis of those EOCs with partial deletion suggests at least three distinct regions of allele imbalance, at 11p15.5–p15.3, 11q23.3–24.1 and 11p12–q14.3.

In contrast to previous reports (Zheng et al., 1991; Kiechleschwarz et al., 1993), we found no significant association between differentiation grade and allele imbalance at the 11p15 region (despite 67% LOH) in our sample of EOCs, and this may reflect the lack of uniformity in ovarian cancer grading methodology; nor could we confirm an association between 11p15 LOH and advanced stage disease (Viel et al., 1992), although there appeared to be a non-significant trend for both these parameters (Figure 2). D11S922 LOH (11p15.5) did correlate at a borderline level of significance ($P = 0.046$) with LOH at D11S935. (Significant LOH rates have been detected in several studies at 11p13, near the site of *WT1*, although direct analysis of the *WT1* locus suggests that it is not the gene involved; Bruening et al., 1993; Viel et al., 1994.) There was also evidence of significant LOH at D11S922 in benign and borderline tumours. With this high level of loss in EOCs, and without significant correlations with advanced disease/poor prognosis subgroups, the likelihood is that an 18.6 Mb interval within 11p15 houses a gene involved at an early stage in ovarian carcinogenesis, occurring as part of the development of benign and borderline tumours and also detectable at roughly similar rates in adenocarcinoma. Allele loss at D11S569 (11p15.3) is low (12.5%) in benign and borderline tumours, but is much higher in carcinomas, and there is no difference in LOH rate between early and advanced FIGO stage adenocarcinomas. This raises the possibility of a second locus at 11p15.3 which is inactivated as part of the development of frank adenocarcinoma (albeit at an early stage of adenocarcinoma development).

We have confirmed and extended (in both numbers and chromosomal position) the recent finding of extensive allele loss distal to 11q23.3 (Foulkes et al., 1993), with 67% of EOCs exhibiting LOH at 11q23.3–24.1 in our sample. Our proximal marker (D11S925) in this region maps about 1.2 Mb telomeric to the most distal marker in the study of Foulkes et al. Loss of heterozygosity at the distal MSP (D11S912) at 11q24.1 is significantly associated with adverse survival and advanced stage, although the latter *P*-value, at 0.035, is borderline.

No borderline tumours exhibited allele imbalance at D11S912. Allele imbalance at the subtelomeric region at D11S912 showed significant correlation with D11S897 (11q23.1) and D11S935 (11p13 in the region of *WT1*). These findings suggest that a TSG acting primarily as a 'progression suppressor' may be located at 11q23.3–24.1 (or telomeric), and that its inactivation may be a significant late event in the pathogenesis of epithelial ovarian cancer.

The 11p12–q14.3 region (which is a large region containing the centromeric half of 11q), although exhibiting high levels of loss, does not appear to segregate significantly with any particular parameter, although there is a non-significant trend towares LOH in association with better prognosis tumours. Allele imbalance in this region does, however, segregate significantly with imbalance of the 11q subtelomeric region. D11S873 LOH at 11q14–q22 seems to correlate with favourable clinicopathological parameters: higher LOH rates are observed in those with mucinous histology, early FIGO stage and well/moderately differentiated tumours. Higher rates of allele loss at D11S873 are seen in those patients remaining alive (also seen at the neighbouring locus, D11S905 at 11p13–12). These findings suggest the possibility that some well-differentiated, early FIGO stage carcinomas may belong to a genetically distinct subcategory of EOC rather than being simply precursors of aggressive late-stage disease (Figure 7), and that allele loss in the 11p12–q22 region may confer changes incompatible with rapid progression of the disease, e.g. deletion of an oncogene locus essential for tumour progression. It is possible that LOH detected in this region could reflect amplification of the 11q13 region similar to that observed in breast cancer (Karlseder et al., 1994), and we have not ruled out this possibility in the present study, although previous studies of this region in ovarian cancer (Foulkes et al., 1993) suggest that

**Figure 7** Hypothesis for chromosome 11 involvement in ovarian carcinogenesis. For abbreviations, see text.

amplification occurs infrequently. However, the explanation for our findings of better prognosis associated with allele loss at this locus remains obscure and requires further work.

The absence of LOH at D11S873 and D11S35 specifically in endometrioid adenocarcinoma is of considerable interest, though the finding approached statistical significance only for the latter MSP ($P = 0.04$). D11S35 lies about 160 kb from the site of the progesterone receptor (PgR) gene. At least six studies have reported that endometrioid tumours contain PgR levels that are elevated relative to other histological types (Slotman and Rao, 1988). Furthermore, there is evidence that, in breast cancer, gene dosage, although secondary to regulatory change, plays a significant role in determining hormone receptor levels: tumours that are cytogenetically 6q−/11q− have half as many oestrogen receptors (ER) and PgRs as tumours without losses on 6q or 11q (Magdelenat *et al.*, 1994). We would therefore speculate that there may be a role for the PgR gene in the regulation of histological subtypes of ovarian cancer, and possibly that its structural disruption contributes to the generation of adverse histological and prognostic subtypes at a relatively early stage in the development of ovarian cancer.

The findings in this study extend the previous observations of distinctive patterns of aneusomy or molecular aberrations in ovarian cancers belonging to different clinicopathological subgroups. They do not imply that LOH at each of the defined regions of chromosome 11 represents independent prognostic factors, although 11q subtelomere imbalance should perhaps be subjected to a large prospective study.

Of more immediate relevance is the application of the observed correlations synthesis of a multistep model of ovarian carcinogenesis (Figure 7). In this model, there is not only multistep, but also multipath, progression of ovarian cancer, postulating, for example, the involvement of q14.3–q22 imbalance in a biologically distinct subpopulation of early EOC.

Extensive chromosome 17 microsatellite and restriction fragment length polymorphism (RFLP) analysis of this same cohort of patients has been performed in our laboratory (Eccles *et al.*, 1992*b*; Steel *et al.*, 1994), and we are currently extending our analysis to correlations between regions of LOH on chromosomes 11 and 17 and their relationship to clinicopathological parameters. In addition, more detailed mapping of LOH within the 11q23.3–qter region to better define the imbalance peak is in progress. This will also determine if there are regions telomeric to 11q24 with low rates of LOH and address the possibility that these observed losses are simply due to non-specific telomeric high-frequency breakages as a consequence of neoplasia rather than putative tumour-suppressor genes at these sites.

**Acknowledgements**
We are grateful to Mr Spike Clay, MRC Human Genetics Unit, for technial assistance and Ms Sharon Love, ICRF Medical Statistics Laboratory, for constructively critical statistical advice.

### References

AALTONEN LA, PELTOMAKI P, LEACH FS, SISTONEN P, PYLKKANEN L, MECKLIN JP, JARVINEN H, POWELL SM, JEN J, HAMILTON SR, PETERSEN GM, KINZLER KW, VOGELSTEIN B AND DE LA CHAPELLE A. (1993). Clues to the pathogenesis of familial colorectal cancer. *Science*, **260**, 812–816.

BELLO MJ AND REY JA. (1990). Chromosome aberrations in metastatic ovarian cancer: relationship with abnormalities in primary tumours. *Int. J. Cancer*, **45**, 50–54.

BRUENING W, GROS P, SATO T, STANIMIR J, NAKAMURA Y, HOUSMAN D AND PELLETIER J. (1993). Analysis of the 11p13 Wilms tumor suppressor gene (wt1) in ovarian tumors. *Cancer Invest.*, **11**, 393–399.

CALL KM, GLASER T, ITO CY, BUCKLER AJ, PELLETRIER J, HABER DA, ROSE EA, KRAL A, YEGER H, LEWIS WH, JONES C AND HOUSEMAN DE. (1990). Isolation and characterisation of a zinc finger polypeptide gene at the human chromosome 11 Wilms' tumor locus. *Cell*, **60**, 509–520.

COHEN BB, WALLACE MR AND CRICHTON DN. (1992). A comparison of procedures for analysing microsatellite repeat polymorphisms. *Mol. Cell. Probes*, **6**, 439–442.

COUILLIN P, LEGUERN E, VIGNAL A, FIZAMES C, RAVISE N, DELPORTES D, REGUIGNE I, ROSIER M, JUNIEN C, VANHEYNINGEN V AND WEISSENBACH J. (1994). Assignment of 112 microsatellite markers to 23 chromosome-11 subregions delineated by somatic hybrids – comparison with the genetic map. *Genomics*, **21**, 379–387.

ECCLES D, CRANSTON G, STEEL CM, NAKAMURA Y AND LEONARD RCF (1990). Allele losses on chromosome 17 in human epithelial ovarian cancer. *Oncogene*, **5**, 1599–1601.

ECCLES DM, GRUBER L, STEWART M, STEEL CM AND LEONARD RCF. (1992*a*). Allele loss on chromosome-11p is associated with poor survival in ovarian cancer. *Dis. Markers*, **10**, 95–99.

ECCLES DM, RUSSELL S, HAITES NE, ATKINSON R, BELL DW, GRUBER L, HICKEY I, KELLY K, KITCHENER H AND LEONARD R. (1992*b*). Early loss of heterozygosity on 17q in ovarian cancer. *Oncogene*, **7**, 2069–2072.

EHLEN T AND DUBEAU L. (1990). Loss of heterozygosity on chromosomal segments 3p, 6q and 11p in human ovarian cancer. *Oncogene*, **5**, 219–223.

FOULKES WD, CAMPBELL IG, STAMP G AND TROWSDALE J. (1993). Loss of heterozygosity and amplification on chromosome 11q in human ovarian cancer. *Br. J. Cancer*, **67**,, 268–273.

FUTREAL P, SODERKVIST P, MARKS JR, IGLEHART JD, COCHRAN C, BARRETT JC AND WISEMAN RW. (1992). Detection of frequent allelic loss on proximal chromosome 17 in sporadic breast carcinoma using microsatellite length polymorphisms. *Cancer Res.*, **52**, 2624–2627.

GALLION HH, POWELL DE, MORROW JK, PIERETTI M, CASE E, TURKER MS, DEPRIEST PD, HUNTER JE AND VANNAGELL JR. (1992). Molecular genetic changes in human epithelial ovarian malignancies. *Gynecol. Oncol.*, **47**, 137–142.

GYAPAY G, MORISSETTE J, VIGNAL A, DIB C, FIZAMES C, MILLASSEAU P, MARC S, BERNARDI G, LATHROP M AND WEISSENBACH J. (1994). The 1993–94 Genethon human genetic linkage map. *Nature Genet.*, **7**, 246–300.

JAMES MR, RICHARD III CW, SCHOTT J-J, YOUSTRY C, CLARK K, BELL J, TERWILLIGER JD, HAZAN J, DUBAY C, VIGNAL A, AGRAPART M, IMAI T, NAKAMURA Y, POLYMEROPOULOUS M, WEISSENBACH J, COX DR AND LATHROP GM. (1994). A radiation hybrid map of 506 STS markers spanning human chromosome 11. *Nature Genet.*, **8**, 70–76.

JENKINS RB, BARTELT D, STALBOERGER P, PERSONS D, DAHL RJ, PODRATZ K, KEENEY G AND HARTMANN L. (1993). Cytogenetic studies of epithelial ovarian carcinoma. *Cancer Genet. Cytogenet.*, **71**, 76–86.

KARLSEDER J, ZEILLINGER R, SCHNEEBERGER C, CZERWENKA K, SPEISER P, KUBISTA E, BIRNBAUM D, GAUDRAY P AND THEILLET C. (1994). Patterns of DNA amplification at band-q13 of chromosome-11 in human breast-cancer. *Genes Chrom. Cancer*, **9**, 42–48.

KIECHLESCHWARZ M, BAUKNECHT T, WIENKER T, WALZ L AND PFLEIDERER A. (1993). Loss of constitutional heterozygosity on chromosome-11p in human ovarian-cancer – positive correlation with grade of differentiation. *Cancer*, **72**, 2423–2432.

LEE JH, KAVANAGH JJ, WHARTON JT, WILDRICK DM AND BLICK M. (1989). Allele loss at the c-Ha-ras1 locus in human ovarian cancer. *Cancer Res.*, **49**, 1220–1222.

LI SB, SCHWARTZ PE, LEE WH AND YANGFENG TL. (1991). Allele loss at the retinoblastoma locus in human ovarian cancer. *J. Natl Cancer Inst.*, **83**, 637–640.

LITT M, KRAMER P, HAUGE XY, WEBER JL, WANG Z, WILKIE PJ, HOLT MS, MISHRA S, DONISKELLER H AND WARNICH L. (1993). A microsatellite-based index map of human chromosome-11. *Hum. Mol. Genet.*, **2**, 909–913.

MAGDELENAT H, GERBAULT-SEUREAU M AND DUTRILLAUX B. (1994). Relationship between loss of estrogen and progesterone receptor expression and of 6q and 11q chromosome arms in breast cancer. *Int. J. Cancer*, **57**, 63–66.

PEJOVIC T, HEIM S, MANDHAL N, BALDETORP B, ELMFORS B, FLODERUS U-M, FURGYIK S, HELM G, HIMMELMAN A, WILLEN H & MITELMAN F. (1992). Chromosome aberration in 35 primary ovarian carcinomas. *Genes Chrom. Cancer*, **4**, 58–68.

PONDER B. (1988). Gene losses in human tumours. *Nature*, **335**, 400–402.

SATO T, SAITO H, MORITA R, KOI S, LEE JH AND NAKAMURA Y. (1991). Allelotype of human ovarian cancer. *Cancer Res.*, **51**, 5118–5122.

SLOTMAN BJ AND RAO BR. (1988). Ovarian cancer (review). *Anticancer Research*, **8**, 417–434.

STEEL CM, ECCLES DM, GRUBER L, WALLACE M, LESSELS A., MORSMAN JM, GABRA H, LEONARD RCF, AND COHEN BB. (1994). Allele losses on chromosome 17 in ovarian tumours. In *Ovarian Cancer*, Vol. 3, Sharp F, Mason P, Blackett T and Berek J (eds) pp. 45–52. Chapman & Hall Medical: London.

THIBODEAU SN, BREN G AND SCHAID D. (1993). Microsatellite instability in cancer of the proximal colon. *Science*, **260**, 816–819.

VANDAMME B, LISSENS W, AMFO K, DESUTTER P, BOURGAIN C, VAMOS E AND DEGREVE J. (1992). Deletion of chromosome 11p13–11p15.5 sequences in invasive human ovarian cancer is a subclonal progression factor. *Cancer Res.*, **52**, 6646–6652.

VIEL A, GIANNINI F, TUMIOTTO L, SOPRACORDEVOLE F, VISENTIN MC AND BOIOCCHI M. (1992). Chromosomal localization of 2 putative 11p oncosuppressor genes involved in human ovarian tumors. *Br. J. Cancer*, **66**, 1030–1036.

VIEL A, GIANNINI F, CAPOZZI E, CANZONIERI V, SCARABELLI C, GLOGHINI A AND BOIOCCHI M. (1994). Molecular mechanisms possibly affecting wt1 function in human ovarian tumors. *Int. J. Cancer*, **57**(4), 515–521.

WEISSENBACH J, GYAPAY G, DIB C, VIGNAL A, MORISSETTE J, MILLASSEAU P, VAYSSEIX G AND LATHROP M. (1992). A second-generation linkage map of the human genome. *Nature*, **359**, 794–801.

YANGFENG TL, LI SB, HAN H AND SCHWARTZ P. (1992). Frequent loss of heterozygosity on chromosome-xp and chromosome-13q in human ovarian cancer. *Int. J. Cancer*, **52**, 575–580.

ZHENG JP, ROBINSON WR, EHLEN T, YU MC AND DUBEAU L. (1991). Distinction of low-grade from high-grade human ovarian carcinomas on the basis of losses of heterozygosity on chromosome-3, chromosome-6, and chromosome-11 and her-2/neu gene amplification. *Cancer Res.*, **51**, 4045–4051.